# Characteristics Associated with Hospitalization Among Patients with COVID-19 — Metropolitan Atlanta, Georgia, March–April 2020

**DOI:** 10.15585/mmwr.mm6925e1

**Published:** 2020-06-26

**Authors:** Marie E. Killerby, Ruth Link-Gelles, Sarah C. Haight, Caroline A. Schrodt, Lucinda England, Danica J. Gomes, Mays Shamout, Kristen Pettrone, Kevin O'Laughlin, Anne Kimball, Erin F. Blau, Eleanor Burnett, Chandresh N. Ladva, Christine M. Szablewski, Melissa Tobin-D’Angelo, Nadine Oosmanally, Cherie Drenzek, David J. Murphy, James M. Blum, Julie Hollberg, Benjamin Lefkove, Frank W. Brown, Tom Shimabukuro, Claire M. Midgley, Jacqueline E. Tate, CDC COVID-19 Response Clinical Team, CDC COVID-19 Response Clinical Team, Sean D. Browning, Beau B. Bruce, Juliana da Silva, Jeremy A.W. Gold, Brendan R. Jackson, Sapna Bamrah Morris, Pavithra Natarajan, Robyn Neblett Fanfair, Priti R. Patel, Jessica Rogers-Brown, John Rossow, Karen K. Wong

**Affiliations:** ^1^CDC COVID-19 Emergency Response Team; ^2^Epidemic Intelligence Service, CDC; ^3^Georgia Department of Public Health; ^4^Emory University School of Medicine, Atlanta, Georgia; ^5^Emory Decatur Hospital, Decatur, Georgia.; CDC; CDC; CDC; CDC; CDC; CDC; CDC; CDC; CDC; CDC; CDC; CDC.

*On June 17, 2020, this report was posted online as an *MMWR* Early Release.*

The first reported U.S. case of coronavirus disease 2019 (COVID-19) was detected in January 2020 (*1*). As of June 15, 2020, approximately 2 million cases and 115,000 COVID-19–associated deaths have been reported in the United States.* Reports of U.S. patients hospitalized with SARS-CoV-2 infection (the virus that causes COVID-19) describe high proportions of older, male, and black persons (*2*–*4*). Similarly, when comparing hospitalized patients with catchment area populations or nonhospitalized COVID-19 patients, high proportions have underlying conditions, including diabetes mellitus, hypertension, obesity, cardiovascular disease, chronic kidney disease, or chronic respiratory disease (*3*,*4*). For this report, data were abstracted from the medical records of 220 hospitalized and 311 nonhospitalized patients aged ≥18 years with laboratory-confirmed COVID-19 from six acute care hospitals and associated outpatient clinics in metropolitan Atlanta, Georgia. Multivariable analyses were performed to identify patient characteristics associated with hospitalization. The following characteristics were independently associated with hospitalization: age ≥65 years (adjusted odds ratio [aOR] = 3.4), black race (aOR = 3.2), having diabetes mellitus (aOR = 3.1), lack of insurance (aOR = 2.8), male sex (aOR = 2.4), smoking (aOR = 2.3), and obesity (aOR = 1.9). Infection with SARS-CoV-2 can lead to severe outcomes, including death, and measures to protect persons from infection, such as staying at home, social distancing (*5*), and awareness and management of underlying conditions should be emphasized for those at highest risk for hospitalization with COVID-19. Measures that prevent the spread of infection to others, such as wearing cloth face coverings (*6*), should be used whenever possible to protect groups at high risk. Potential barriers to the ability to adhere to these measures need to be addressed.

Patients were selected from six acute care hospitals and associated outpatient clinics affiliated with a single academic health care system in metropolitan Atlanta. Hospitalized patients were selected sequentially from hospital-provided lists of patients aged ≥18 years who were hospitalized with laboratory-confirmed COVID-19 (defined as a positive real-time reverse transcription–polymerase chain reaction [RT-PCR] test result for SARS-CoV-2) during March 1–30. The 220 selected hospitalized patients were described previously (*2*); hospitalizations included stays for observation and deaths that occurred in an emergency department (ED). All 311 nonhospitalized patients (i.e., evaluated at outpatient clinics or an ED and not admitted) aged ≥18 years with laboratory-confirmed COVID-19 during March 1–April 7, were included, unless they stayed for observation or died in an ED. During April 8–May 1, trained personnel abstracted information from electronic medical records on patient demographics, occupation, underlying conditions, and symptoms using REDCap software (version 8.8.0; Vanderbilt University) (*7*). This investigation was determined by CDC to be public health surveillance and by the Georgia Department of Public Health as an institutional review board–exempt public health evaluation.

During March 1–April 7, 2020, the health care system operated a telephone triage line to manage incoming patients with COVID-19–compatible symptoms. Patients with signs of severe illness (e.g., severe shortness of breath, confusion, or hemoptysis) were directed to an ED. Other symptomatic persons could receive outpatient SARS-CoV-2 testing; however, testing was limited, and appointments were prioritized for health care personnel and persons considered to be at higher risk for severe COVID-19–associated illness (e.g., persons aged ≥65 years and those with underlying conditions, including diabetes mellitus, cardiovascular disease, and chronic respiratory disease).

For analyses, race was categorized as black or other race; obesity was defined as body mass index ≥30 kg/m^2^; age was categorized as 18–44, 45–64, and ≥65 years; smoking was defined as being a current or former smoker; cardiovascular disease excluded hypertension alone; and chronic kidney disease included end stage renal disease. Health care personnel were classified as persons whose occupations included patient contact or possible exposure to infectious agents in a health care setting.^†^ Univariable and multivariable logistic regressions were used to compare hospitalized with nonhospitalized patients; variables included age group, race, sex, smoking status, insurance status, obesity, hypertension, diabetes mellitus, cardiovascular disease, chronic respiratory disease, and chronic kidney disease. These variables were selected based upon risk factors for severe COVID-19 identified in other studies (*3*,*4*) rather than a defined statistical endpoint. Persons lacking a health care visit during which a medical history could be recorded (25) were excluded from analyses. Because of small sample sizes for some variables, Firth’s correction was used to provide bias-reduction (*8*). Because information on race was missing for nearly one quarter (23%) of nonhospitalized patients, sensitivity analyses were performed. Multivariable analyses were repeated and any patient with missing race was reclassified, first as black, then as other race. This method of sensitivity analysis was used to avoid implicit assumptions about the nature of missing data. Data were analyzed using SAS statistical software (version 9.4; SAS Institute).

Compared with nonhospitalized patients (311), hospitalized patients (220) were older (median age = 61 years) and more frequently male (52%) and black (79%) (Table). Obesity, smoking, hypertension, diabetes mellitus, and chronic kidney disease were more prevalent among hospitalized patients than among nonhospitalized patients. Among those whose occupations were reported, nonhospitalized patients were more likely to be health care personnel (54%) than were hospitalized patients (4%). Fever or cough were commonly reported among both hospitalized and nonhospitalized patients, whereas shortness of breath was reported more often among hospitalized patients. Chills, headache, loss of smell or taste, or sore throat were reported more often among nonhospitalized patients.

**TABLE:** Characteristics of hospitalized and nonhospitalized patients with COVID-19 treated at six acute care hospitals and associated outpatient clinics in metropolitan Atlanta, Georgia, March 1–April 7, 2020

Demographic characteristic	No. (%) of patients	
	Nonhospitalized (n = 311)	Hospitalized (n = 220)
**Sex**		
Male	114 (36.7)	114 (51.8)
Female	197 (63.3)	106 (48.2)
**Age group (yrs)**		
**Median age, yrs (IQR)**	45.0 (33.0–58.0)	61.0 (45.0–70.0)
18–44	151 (48.6)	54 (24.6)
45–64	120 (38.6)	76 (34.6)
≥65 years	40 (12.9)	90 (40.9)
**Race**		
White	90 (28.9)	29 (13.2)
Black	139 (44.7)	174 (79.1)
Other	10 (3.2)	7 (3.2)
Missing race	72 (23.2)	10 (4.6)
**Ethnicity**		
Hispanic	10 (3.2)	6 (2.7)
Non-Hispanic*	197 (63.3)	203 (92.3)
Missing ethnicity	104 (33.4)	11 (5.0)
**Occupation**		
Health care personnel^†^	168 (54.0)	8 (3.6)
Non-health care personnel	78 (25.1)	50 (22.7)
Missing occupation	65 (20.9)	162 (73.6)
**Other characteristic**		
Uninsured	20 (6.4)	22 (10.0)
Missing insurance status	6 (1.9)	3 (1.4)
Lives in a congregate living facility^§^	1 (0.3)	12 (5.5)
Pregnant	4 (1.3)	3 (1.4)
Past or current smoking	37 (11.9)	54 (24.6)
Missing smoking status	52 (16.7)	9 (4.1)
**Underlying condition**		
Obesity^¶^	104 (33.4)	123 (55.9)
Missing BMI	84 (27.0)	11 (5.0)
Cardiovascular disease	12 (3.9)	8 (3.6)
Hypertension	101 (32.5)	142 (64.6)
Diabetes mellitus	30 (9.7)	81 (36.8)
Type 1	2 (0.6)	2 (0.9)
Type 2	28 (9.0)	74 (33.6)
Chronic respiratory disease	56 (18.0)	45 (20.5)
Chronic kidney disease	7 (2.3)	38 (17.3)
Chronic kidney disease without dialysis	6 (1.9)	24 (10.9)
End stage renal disease	1 (0.3)	14 (6.4)
Any transplant	1 (0.3)	10 (4.6)
Liver disease	4 (1.3)	5 (2.3)
HIV infection	10 (3.2)	5 (2.3)
Cancer	28 (9.0)	6 (2.7)
Rheumatological disease	4 (1.3)	6 (2.7)
No. of underlying conditions**		
0	169 (54.3)	44 (20.0)
1	88 (28.3)	77 (35.0)
2	44 (14.2)	65 (29.6)
≥3	10 (3.2)	34 (15.5)
**Symptoms at initial evaluation**		
Fever^††^	240 (77.2)	188 (85.5)
Cough	275 (88.4)	180 (81.8)
Shortness of breath (dyspnea)	135 (43.4)	149 (67.7)
Headache	171 (55.0)	35 (15.9)
Chills	178 (57.2)	58 (26.4)
Arthralgia	44 (14.2)	9 (4.1)
Myalgia	184 (59.2)	69 (31.4)
Sore throat	146 (47.0)	21 (9.6)
Loss of smell^§§^	130 (41.8)	4 (1.8)
Loss of taste	106 (34.1)	6 (2.7)
Gastrointestinal symptoms^¶¶^	137 (44.1)	88 (40.0)
**Median interval between symptom onset and testing, days (IQR)**	**4.0 (2.0–7.0)**	**6.0 (3.0–9.5)**

**Abbreviations:** BMI = body mass index; HIV = human immunodeficiency virus; IQR = interquartile range.

* Includes non-Hispanic white and other races/ethnicities.

^†^ Includes any occupation with patient contact.

^§^ Includes nursing homes, assisted living facilities, shelters, and dormitories.

^¶^ BMI ≥30.0 kg/m^2^.

** Includes cardiovascular disease, hypertension, diabetes, chronic respiratory disease, and chronic kidney disease.

^††^ Includes subjective or objective fever (≥100.4°F [38°C]).

^§§^ Loss of smell or taste was first widely reported on April 23, 2020; differences in the periods of investigations between hospitalized and nonhospitalized patients might be responsible for differences in proportions reported.

^¶¶^ Includes abdominal pain, diarrhea, nausea, or vomiting.

After controlling for age, sex, race, obesity, smoking status, insurance status, hypertension, diabetes mellitus, cardiovascular disease, chronic respiratory disease, and chronic kidney disease, characteristics independently associated with hospitalization were age ≥65 years (aOR = 3.4, 95% confidence interval [CI] = 1.6–7.4); black race (aOR = 3.2, 95% CI = 1.8–5.8); having diabetes mellitus (aOR = 3.1, 95% CI = 1.7–5.9); lack of insurance (aOR = 2.8, 95% CI 1.1–7.3); male sex (aOR = 2.4, 95% CI = 1.4–4.1); smoking (aOR = 2.3, 95% CI = 1.2–4.5); and obesity (aOR = 1.9, 95% CI = 1.1–3.3) (Figure). When missing race was reclassified as black or other race in sensitivity analyses, associations with hospitalization did not appreciably change for any variables.

**FIGURE:**
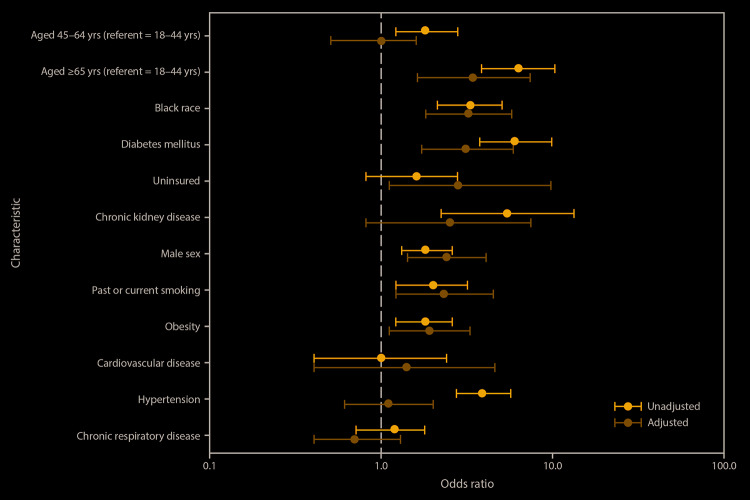
Unadjusted and adjusted* odds ratios and 95% confidence intervals for hospitalizations in COVID-19 patients (n = 506^†^) evaluated at six acute care hospitals and associated outpatient clinics, by selected characteristics — metropolitan Atlanta, Georgia, March 1–April 7, 2020 **Abbreviation:** COVID-19 = coronavirus disease 2019. * Adjusted for age, sex, race, obesity, past or current smoking, insurance status, obesity, and other underlying conditions (hypertension, diabetes mellitus, cardiovascular disease, chronic respiratory disease, and chronic kidney disease). ^†^ Complete case analysis was used for multivariable analyses; therefore, n = 368 for the multivariable model.

## Discussion

Older age, as measured by age ≥65 years, was associated with hospitalization, consistent with previous findings (*3*,*4*). Hospitalized patients with COVID-19 were more likely to have diabetes mellitus and obesity than were nonhospitalized patients, suggesting a relationship between these underlying conditions and increased severity of illness. Diabetes mellitus has been determined to be associated with more severe illness in hospitalized patients with COVID-19 (*4*) and in persons with illness caused by Middle East respiratory syndrome coronavirus (*9*). Obesity has previously been reported to be overrepresented in hospitalized patients with COVID-19 (*3*) and associated with hospitalization (*4*). After controlling for other underlying conditions and patient characteristics, hypertension was no longer associated with hospitalization, suggesting that other underlying conditions or factors associated with hypertension might be partially responsible for the higher prevalence of hypertension in hospitalized COVID-19 patients.

The COVID-19 pandemic has highlighted persistent health disparities in the United States. In a previous investigation of hospitalized patients in Georgia, including the subset of hospitalized patients reported here, the proportion of patients who were black was higher than expected based on overall hospitalizations during the same period (*2*). Racial and ethnic minority groups are at higher risk for severe complications from COVID-19 because of the increased prevalence of diabetes, cardiovascular disease, and other underlying conditions among racial and ethnic minority groups.^§^ Social determinants of health might also contribute to the disproportionate incidence of COVID-19 in racial and ethnic minority groups, including factors related to housing, economic stability, and work circumstances.^¶^ In the United States, black workers are more likely than other workers to be frontline industry or essential workers,** which increases their likelihood of infection with SARS-CoV-2 while performing their jobs. This and other social factors could contribute to the disproportionate diagnoses of COVID-19 among black persons in metropolitan Atlanta.

Black race has previously been associated with increased hospitalization among COVID-19 patients (*10*); however, race has not been associated with mortality among patients who were hospitalized (*2*,*10*). The independent association between black race and hospitalization in this investigation remained, even when the analysis controlled for other characteristics (including diagnosed underlying conditions), suggesting underlying conditions alone might not account for the higher rate of hospitalization among black persons. This might indicate that black persons are more likely to be hospitalized because of more severe illness, or it might indicate that black persons are less likely to be identified in the outpatient setting, potentially reflecting differences in health care access or utilization or other factors not identified through medical record review. Additional research is needed to more fully understand the association between black race and hospitalization. CDC and state and local partners are working to ensure completeness of race and ethnicity data and will continue to analyze and report on racial and ethnic disparities to further elucidate factors and health disparities associated with COVID-19 incidence and illness severity.

The findings in this report are subject to at least five limitations. First, although this investigation identified COVID-19 patients from a single health care system, hospitalized patients likely represent a broader population than nonhospitalized patients because those experiencing mild illness might have accessed outpatient services outside of this health care system or chosen not to seek care. Differences in these two populations caused by selection bias might therefore result in nonhospitalized patients differing beyond having milder illness than hospitalized patients. Thus, in this report, hospitalization status might not only represent severity of illness but also care seeking and potentially other confounding characteristics. Second, given that outpatient testing was prioritized for certain persons, older patients and those with underlying conditions might be overrepresented among outpatients receiving testing, resulting in underestimated odds ratios for hospitalization. In addition, overrepresentation of health care personnel in the outpatient setting could result in overestimation of odds ratios if health care personnel were disproportionately young or healthy. Third, outpatient visits did not always include a full medical history; thus, underlying conditions and other characteristics might be underreported. Fourth, data on age was stratified into groups, and because of sample size, smaller age group categories could not be explored. Finally, data on race, body mass index, and smoking status were missing for a substantial proportion of nonhospitalized patients. Data could not be disaggregated for other races or analyzed by ethnicity because of small sample sizes.

This investigation found that age ≥65 years, black race, and having diabetes mellitus were independently associated with hospitalization. Among the underlying conditions included in the multivariable analysis, diabetes mellitus was most strongly associated with hospitalization. The reported association between black race and hospitalization, which remained even after controlling for diagnosed underlying conditions, suggests that underlying conditions alone might not account for the higher rate of hospitalization among black persons. Other factors that might explain higher rates of hospitalization include health care access, other social determinants of health, or the possibility of bias. Infection with SARS-CoV-2 can lead to severe outcomes, including death, and measures to protect persons from infection such as staying at home, social distancing (*5*), and awareness and management of underlying conditions should be emphasized for those at highest risk for hospitalization with COVID-19. To protect groups at high risk, measures that prevent the spread of infection to others, such as wearing cloth face coverings (*6*), should be used whenever possible. Potential barriers to the ability to adhere to these measures need to be addressed.

**Summary**

**What is already known about this topic?**

Hospitalized COVID-19 patients are more commonly older, male, of black race, and have underlying conditions. Less is known about factors increasing risk for hospitalization.

**What is added by this report?**

Data for 220 hospitalized and 311 nonhospitalized COVID-19 patients from six metropolitan Atlanta hospitals and associated outpatient clinics found that older age, black race, diabetes, lack of insurance, male sex, smoking, and obesity were independently associated with hospitalization.

**What are the implications for public health practice?**

To reduce severe outcomes from COVID-19, measures to prevent infection with SARS-COV-2 should be emphasized for persons at highest risk for hospitalization with COVID-19. Potential barriers to the ability to adhere to these measures need to be addressed.

## Footnotes

* https://www.cdc.gov/coronavirus/2019-ncov/cases-in-us.html.

† https://www.cdc.gov/coronavirus/2019-ncov/hcp/infection-control-recommendations.html.

§
https://www.cdc.gov/mmwr/preview/mmwrhtml/su6203a2.htm

¶ https://www.healthypeople.gov/2020/topics-objectives/topic/social-determinants-of-health.

** https://www.epi.org/publication/black-workers-covid/.
